# A novel SNP in *NKX1-2* gene is associated with carcass traits in Dezhou donkey

**DOI:** 10.1186/s12863-023-01145-2

**Published:** 2023-08-07

**Authors:** Xinrui Wang, Tianqi Wang, Huili Liang, Liyuan Wang, Faheem Akhtar, Xiaoyuan Shi, Wei Ren, Bingjian Huang, Xiyan Kou, Yinghui Chen, Yandong Zhan, Changfa Wang

**Affiliations:** Liaocheng Research Institute of Donkey High-Efficiency Breeding and Ecological Feeding, College of Agronomy and Agricultural Engineering, Liaocheng University, Liaocheng, 252059 China

**Keywords:** *NKX1-2*, Dezhou donkey, Body measurement traits, Carcass traits, SNP

## Abstract

**Background:**

At present, donkey meat in the market shows an imbalance between supply and demand, and there is an urgent need to cultivate a meat-type Dezhou donkey breed. On the one hand, it can improve the imbalance in the market, and on the other hand, it can promote the rapid development of the donkey industry. This study aimed to reveal significant genetic variation in the *NK1 homeobox 2* gene (*NKX1-2*) of Dezhou donkeys and investigate the association between genotype and body size in Dezhou donkeys.

**Results:**

In this study, a SNP (g.54704925 A > G) was identified at the exon4 by high-depth resequencing of the Dezhou donkey *NKX1-2* gene. The AA genotype is the dominant genotype. The g.54704925 A > G site was significantly associated with body length, thoracic girth, and hide weight (*P* < 0.05), while it was highly significantly associated with body height and carcass weight (*P* < 0.01) in Dezhou donkeys.

**Conclusion:**

Overall, the results of this study showed that the *NKX1-2* gene could be a candidate gene for breeding meat-type Dezhou donkeys, and the g.54704925 A > G locus could be used as a marker locus for selection and breeding.

**Supplementary Information:**

The online version contains supplementary material available at 10.1186/s12863-023-01145-2.

## Introduction

The Dezhou donkey is listed as one of the five best donkey breeds in China: it has a large body, a fast growth rate, good production characteristics, and stable genetic performance [1]. Donkey meat is tender in texture and delicious, with the advantages of lean meat: less fat, more essential amino acids, and polyunsaturated fatty acids (PUFAs) [2, 3]. It is known as “Dragon meat in the sky, donkey meat on the ground” and has excellent nutritional value. It can lessen the harmful effects of saturated fatty acids on the human circulatory system [1]. At present, the stock of donkeys in China is declining sharply, and the good breeds are seriously degraded, resulting in an imbalance between the supply and demand of donkey products in the market [4, 5]. However, the cost of donkey meat has been rising rapidly, and the gap between market supply and demand has grown to be a significant impediment to the growth of the donkey sector economically [6]. Therefore, addressing the supply-demand gap in the donkey industry market through molecular breeding techniques has emerged as a prominent research area.

Meat production is a quantitative trait that is regulated by multiple genes and is also influenced by external environmental factors [7–9]. A SNP (*CAPN1* c.5709G > C) in the *µ-calpain* gene (*CAPN1*) was discovered by Gill et al. to be substantially correlated with the weight of the hindquarter in Aberdeen Angus-sired beef cattle, with animals of the CC genotype having more tender meat and heavier hindquarters [10]. Meanwhile, the *diacylglycerol acyltransferase 1* gene (*DGAT1*) SNP (*DGAT1* c.6829 A > G) was correlated with sirloin weight after maturation, and the AA genotype had heavier sirloin [10]. Liu et al. found that mutations at the g.18114954 C > T locus on the *nuclear receptor subfamily 6 group A member 1* gene (*NR6A1*) in Dezhou donkeys were significantly associated with carcass weight, and individuals with the TT genotype were considerably heavier than those with the CC genotype (*P* < 0.05) [11]. There are few studies on mutations at the gene locus associated with carcass weight and other body size data in donkeys, and carcass weight is influenced by body height, body length, chest circumference, and the number of thoracolumbar vertebrae [12, 13]. Therefore, it is essential to identify mutation sites associated with body size and carcass weight for high-quality breeding donkey resources with a high meat yield.

The NK homeobox family (NKX family) contains many members that display diverse functions in cell fate determination, nervous system development, and tumorigenesis [14–16]. *NKX1-2*, a member of the NKX family, is expressed in neural mesodermal progenitors early in development and is involved in forming all three germ layers [17–19]. Wang et al. conducted a study in mice and found that *Nkx1-2* gene expression could promote c-Met expression, activating the hepatocyte growth factor/c-Met pathway (c-Met pathway) to stimulate liver regeneration [20]. In addition, NKX1-2, a transcriptional repressor, is essential for activating Brachyury in P19 mouse embryonic carcinoma cells [19]. In adipose biology, NKX1-2 promotes lipogenic differentiation of the bone marrow mesenchymal precursor cell line ST2 and inhibits the differentiation of ST2 cells into osteoblasts [21]. However, the association between *NKX1-2* gene polymorphisms and growth traits has not been reported yet.

In this study, polymorphisms in the *NKX1-2* gene of Dezhou donkeys were studied by targeted sequencing, and its association with carcass weight was investigated. This provides a theoretical basis for the subsequent cultivation of donkeys with high meat production performance.

## Materials and methods

### Moral statement

The experimental animals and procedures used in this experiment were authorized and approved by the Animal Welfare and Ethics Committee of the Institute of Animal Sciences, Liaocheng University (No.LC2019-1). The care of experimental animals and the application of experimental practices were governed by local animal welfare laws, guidelines, and codes.

### Animals and date collection

The 393 healthy adult Dezhou donkeys were sourced from a donkey breeding facility in Dezhou, Shandong Province, China. They were both 2-year-old fattening donkeys and lived in standard feeding conditions with the same feeding and management conditions. The 393 donkeys in this study were identified in the same way as the source of the 396 donkeys in “A Novel A > G Polymorphism in the Intron 1 of LCORL Gene Is Significantly Associated with Hide Weight and Body Size in Dezhou Donkey” [22]. Among them, the NKX1-2 gene was not amplified in three donkeys, so it was excluded. 393 donkeys were slaughtered for the purpose of being sold at market. The body size data was measured using a measuring stick and a tape, such as body height (BH, cm), body length (BL, cm), and thoracic girth (TG, cm). The body size measurement operation referred to Zhang’s method [23]. All measurement operations were performed by the same operator to reduce artificial errors. In this study, donkeys were slaughtered after being knocked unconscious by the electric shock method. After slaughter, a digital electronic scale was used for measuring hide weight (HW, kg) and carcass weight (CW, kg). The number of thoracic vertebrae (TN) and lumbar vertebrae (LN) was identified by the DR system in-vivo detection device. In addition, each Dezhou donkey had a 20 ml blood sample taken from the jugular vein using an EDTA blood collection tube, which was then immediately stored at -20 °C.

### DNA extraction

Utilizing the M5 FlexGen Blood DNA Kit (TIANGEN, Beijing, China), the 393 Dezhou donkeys were used for DNA extraction from whole blood [9]. The spectrophotometer (B500, Metash, Shanghai, China) and 1% agarose gel were used to detect the DNA purity (OD260/OD280) and quality [24]. After that, adjust the working solution purity to 50 ng/µL and store it at -80 °C [25].

### Identification and genotyping of ***NKX1-2*** gene polymorphism

To investigate the genetic variation, 393 gene samples were high-depth resequencing for the *NKX1-2* gene by targeted sequencing genotype testing technology (GBTS). GBTS was powered by Shijiazhuang MOL BREEDING Biotechnology Co., Ltd. (Shijiazhuang, China).

A total of 2773 probes were used in targeted sequencing, and then compared to the Dezhou donkey *NKX1-2* gene reference sequence (Assembly ASM1607732v2; NC_052178.1; GCF_016077325.2), the coverage rate was 100%. SNPs with genotype frequencies of less than 5% in the sequencing results were eliminated.

### SNP validation

To validate the targeted sequencing results, the 6 gene samples were randomly selected for PCR amplification to verify the accuracy of the resequencing technology. Primers (Table 1) were designed using Primer 3 (https://bioinfo.ut.ee/primer3-0.4.0/), and PCR amplification was performed on the Dezhou donkey *NKX1-2* gene sequence (GenBank accession number: NC_052178.1). Primers were produced by BGI Genomics (Wuhan) Co., Ltd. (Beijing, China). The Primer working solution was diluted to 10 µmoL/µL. PCR reaction volume is 20 µL, including 10 µL 2× M5 Hiper plus Taq HiFi PCR Mix (Mei5bio, with blue dye, MF002, Beijing, China), 6.8 µL ddH2O, 0.8 µL of each primer (BGI Genomics, Wuhan, China), 50 ng/µL genomic DNA template 1.6 µL. The PCR amplification was performed with the following cycling parameters: pre-denaturation at 95 °C for 10 min; denaturation at 95 °C for 30 s; annealing at 62 °C for 30 s; extension at 72 °C for 1 min; cycle 35 times; extension at 72 °C for 10 min. After the reaction, the PCR amplification target fragments were detected by 1% agarose gel electrophoresis. The PCR amplification products that passed the test were sent to BGI Genomics Co., Ltd. (Wuhan, China) for Sanger sequencing. The sequencing results were aligned using Chromas software (Version V2.6.5, Technelysium Pty Ltd., Queensland, Australia) to identify potential mutation sites.

**Table 1:** Primer information of the Dezhou donkey *NKX1-2* gene used for PCR.

Primer	Location	Primer Sequences (5’-3’)	Tm (°C)	Product size (bp)
1	Exon 4	F: ATGGAAGAAGCAGAACCCCG	62	536
		R: CAGATCCTTGACCCGCTTTG		

### Statistical analysis

Targeting sequencing results were removed from SNPs with less than 5% genotype frequencies. SHEsis online software [26] was used to calculate allele frequencies and genotype frequencies, and a chi-square test was used to verify that the allele frequencies of the sample population conformed to Hardy-Weinberg equilibrium (HWE). Genetic parameters, including polymorphic homozygosity (Ho), heterozygosity (He), information content (PIC), and adequate allele numbers (Ne), were estimated using the Genetic Diversity Indexes Calculator online software (http://www.msrcall.com/Gdicall.aspx). The Countcodon Program (http://www.kazusa.or.jp/codon/countcodon.html) was used to calculate the target gene codon usage frequency online. The 3D structural model of the protein was predicted using SWISS-MODEL online software (https://swissmodel.expasy.org/).

The Dezhou donkeys used in this study came from the same farm, were all the same age and sex, and had the same feeding conditions and management. Therefore, the general linear model in SPSS 26.0 (Statistical Product and Service Solutions, Version 26.0 Edition, IBM, Armonk, NY, USA) software was used in this study to determine the relationship between genotypes and phenotypic traits in Dezhou donkeys, including body height, body length, thoracic girth, hide weight, carcass weight, thoracic vertebrae, and lumbar vertebrae. The model structure is Yi = µ + a.i. + e, where Yi is the individual phenotype values, µ is the mean value of each trait in the population, as is the fixed factor genotype, and e is the random error [22]. Results were presented as Means ± SE. Multiple comparisons of the associations were based on Bonferroni-corrected *p*-Values.

The additive effects (a), dominant effects (d), and substitution effects (ɑ) of alleles on phenotypic traits in Dezhou donkeys were calculated as follows: a = (AA - GG)/2, d = AG – (GG + AA)/2, ɑ = a + d(p - q), where AA and GG represent the mean values of individuals with purely homozygous genotypes, AG represents the mean value of individuals with heterozygous genotypes, p represents the gene frequency of the A allele, and q represents the gene frequency of the G allele.

## Results

### Genetic polymorphism of ***NKX1-2*** gene in Dezhou donkey

**Fig. 1:**
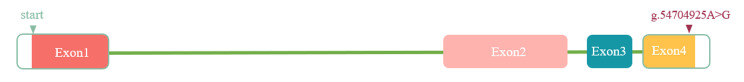
Schematic representation of the *NKX1-2* gene and localization of identified mutation sites. “start” represents the transcription start site of the *NKX1-2* gene, while “g.54704925A > G” represents the mutation site of the *NKX1-2* gene

**Fig. 2:**
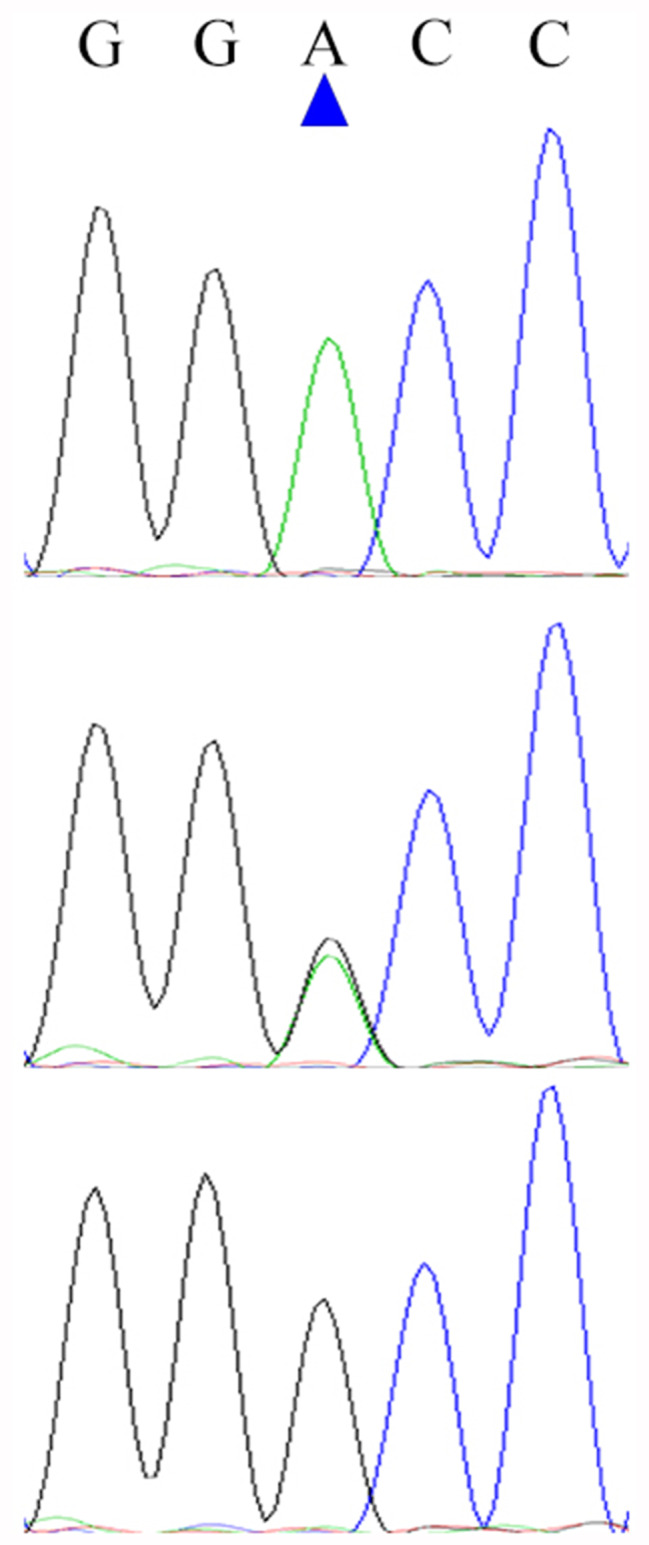
*NKX1-2* gene g.54704925 A > G Sanger sequencing results

**Fig. 3:**
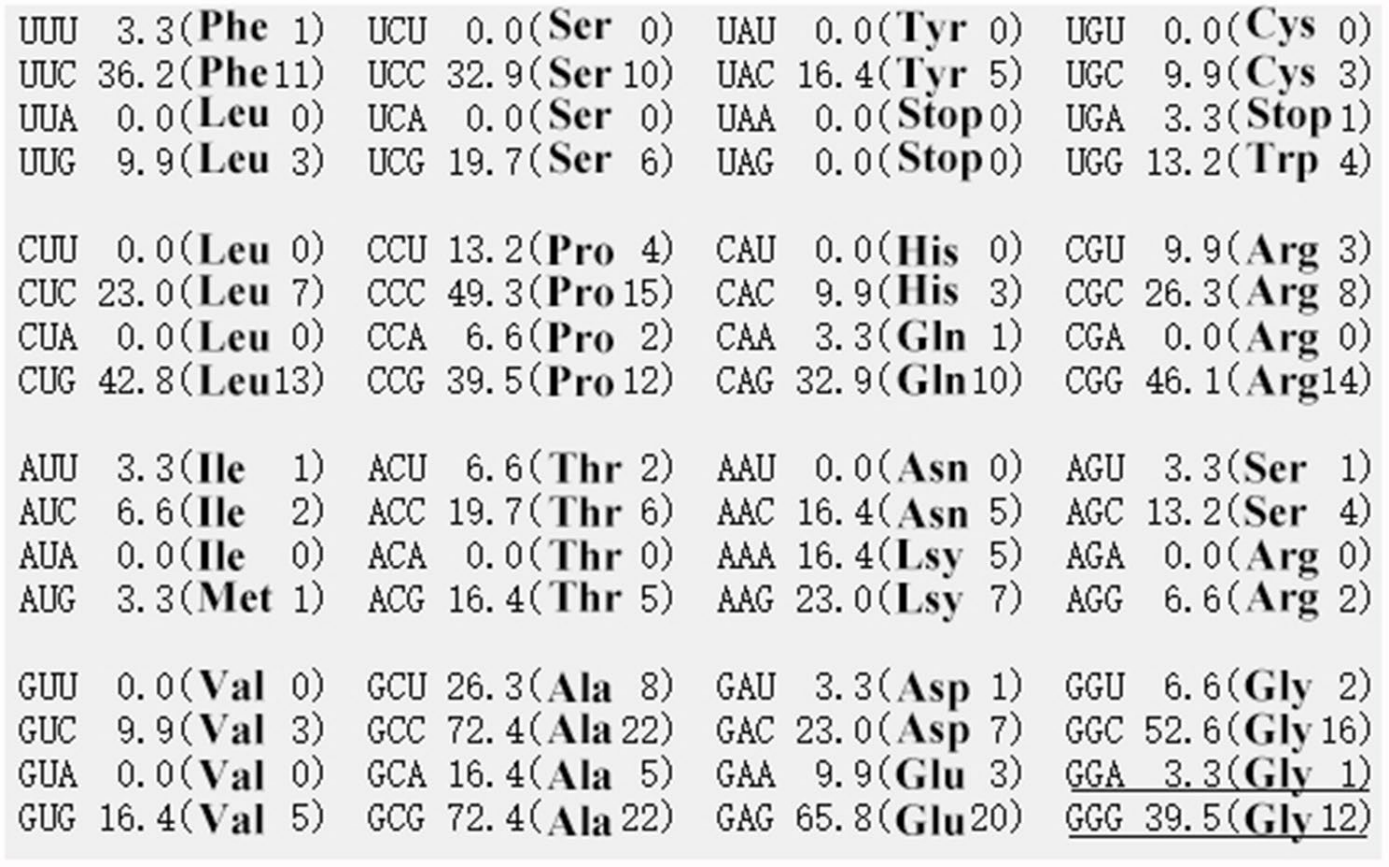
The distribution of genetic codons of Dezhou donkey *NKX1-2 *gene. The underlined bases represent synonymous mutations

**Fig. 4:**
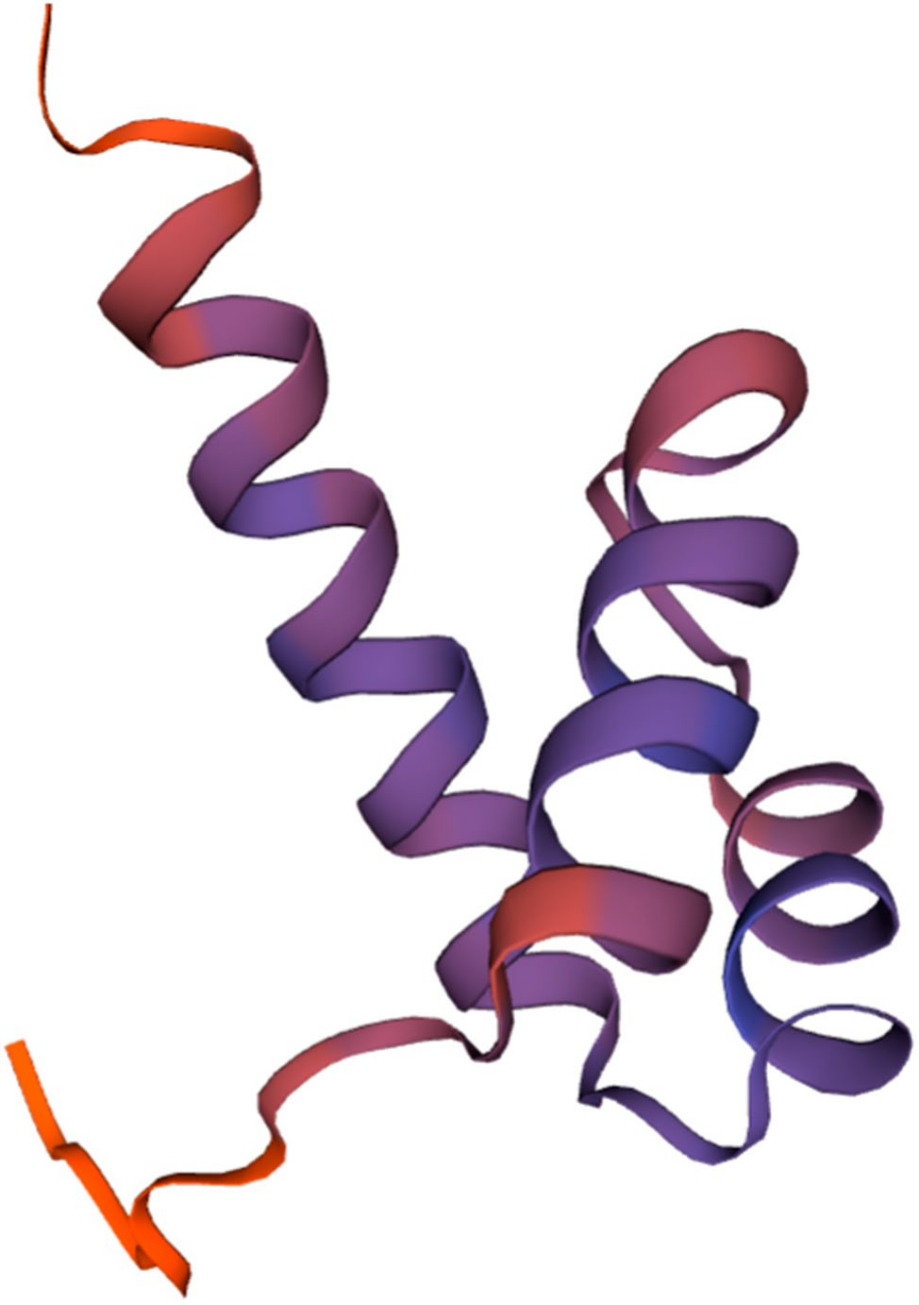
The 3D structural model of the protein of Dezhou donkey *NKX1-2* gene

The Dezhou donkey’s *NKX1-2* gene, which has four exons and three introns, is found on chromosome 2. A total of 32 SNPs were detected by targeted sequencing (Supplementary Table 1), of which 1 was found in the upstream region, 17 in the exon region, 9 in the intron, and 5 in the downstream area. By excluding SNPs with non-diallelic genotypes, genotype frequencies less than 5%, and SNPs that did not conform to HWE (*P* < 0.05), only g.54704925 A > G, located on exon4, was found to be eligible (Fig. 1). The results of Sanger sequencing (Fig. 2) showed that the sequencing results of the mutant site matched the sequencing results of GBTS. g.54704925 A > G is a synonymous mutation that changes codon 291 (Gly) from GGA to GGG. From the frequency of *NKX1-2* gene codon usage (Fig. 3), GGG is the high-frequency codon of Gly, while GGA is the low-frequency codon, indicating the expression level of the *NKX1-2* gene may be affected by the mutation of the g.54704925 A > G locus. Meanwhile, the 3D structural model of the protein encoded by the *NKX1-2* gene is shown in Fig. 4.

### Genetic Parameters of SNP Detected in Dezhou donkey ***NKX1-2*** Gene

The genetic diversity of the *NKX1-2* gene in Dezhou donkeys was evaluated using genotype frequencies, and allele frequencies and four genetic indices (Ho, He, PIC, and Ne) of SNP (Table 2). AA, AG, and GG genotypes were 0.298, 0.501, and 0.201, respectively. The AG genotype had the highest proportion, and the GG genotype had the lowest balance. It indicated that the AG genotype was the dominant genotype in the population. The allele frequencies of A and G were 0.548 and 0.452, respectively, and the A allele was higher than the G allele, indicating that the wild-type allele was dominant in this population. Ho, He, Ne, and PIC values were 0.505, 0.495, 1.982, and 0.353, respectively, moderate polymorphic loci (0.25 < *PIC* < 0.5). This locus is consistent with Hardy-Weinberg equilibrium (HWE) (*P* > 0.05).

**Table 2:** Genotyping and population genetic analysis in *NKX1-2* gene

Position	Location	Sample	Genotype	Genotype Frequencies	Number	Allele Frequencies		HW		Genetic Parameters			
						Wild	Mutant	Chi-Square	p-Value	Ho	He	PIC	Ne
			AA	0.298	117								
g.54704925 A > G	Exon 4	393	AG	0.501	197	0.548	0.452	0.057	0.812	0.505	0.495	0.373	1.982
			GG	0.201	79								

### Association analysis of ***NKX1-2*** gene g.54704925 A > G locus with body measurement traits

**Fig. 5:**
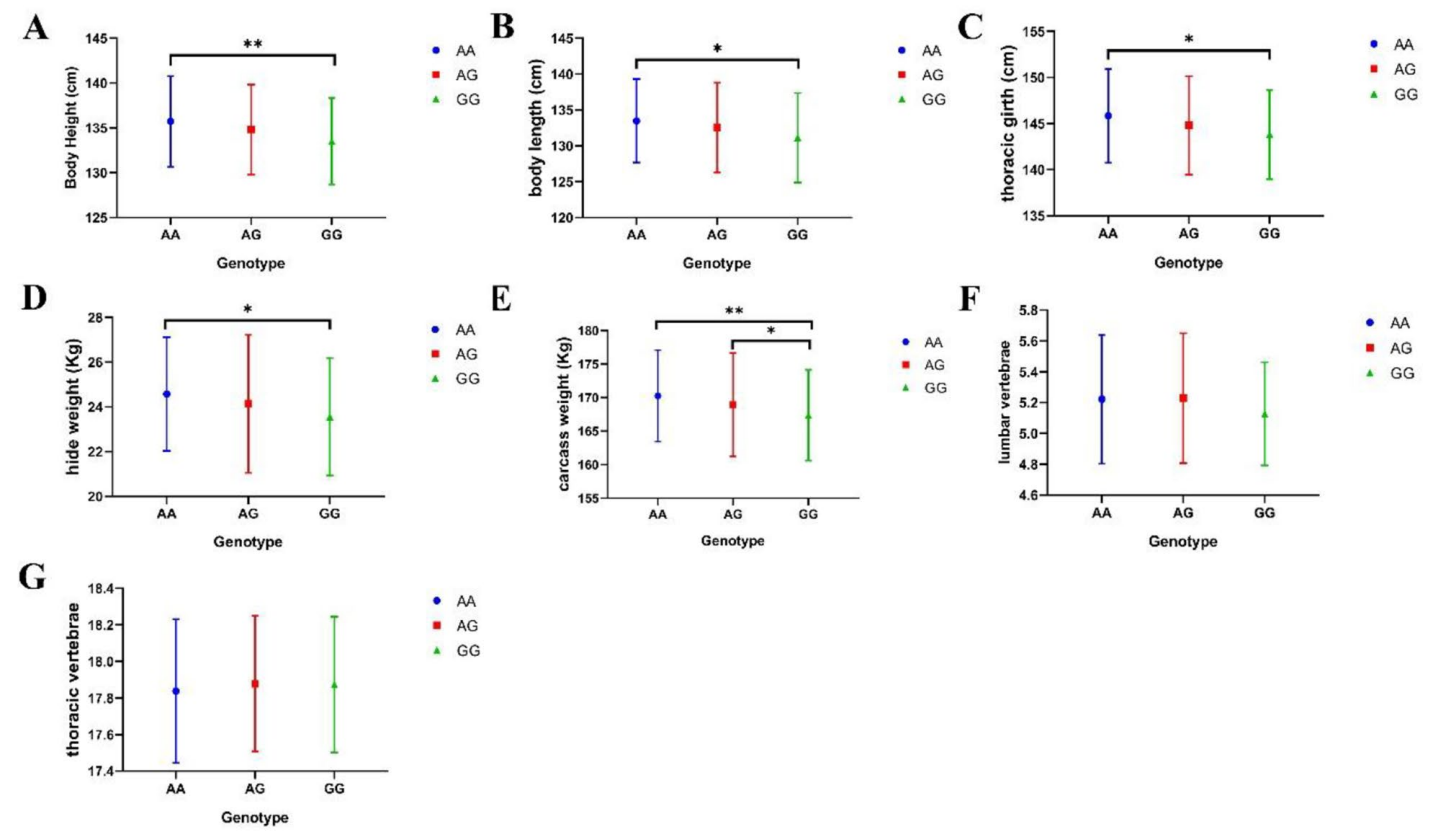
Effect of different genotypes at the g.54704925 A > G locus on traits. * 0.01 < P < 0.05, ** P < 0.01

Association analysis of the *NKX1-2* gene g.54704925 A > G locus with Dezhou donkey body measurement traits was performed, and the results are shown in Table 3; Fig. 5. The results showed that the AA genotype population was significantly higher than the GG genotype population in body length, thoracic girth, and hide weight phenotypes. In contrast, body height and carcass weight phenotypes showed that the AA genotype population was significantly higher than the GG genotype individuals. Interestingly, there were also significant differences between individuals with the AG genotype and GG genotype in the carcass weight phenotype, while no significance was found for all three genotypes in the lumbar vertebrae and thoracic vertebrae phenotypes.

**Table 3:** Association between the g.54704925 A > G locus within the *NKX1-2* gene and body measurement traits of the Dezhou donkey

SNP Site	Genotype/Number	body height (cm)	body length (cm)	thoracic girth (cm)	hide weight (Kg)	carcass weight (Kg)	lumbar vertebrae	thoracic vertebrae
g.54704925 A > G	AA/117	135.72 ± 0.47^a^	133.48 ± 0.54^a^	145.83 ± 0.47^a^	24.58 ± 0.24^a^	155.07 ± 1.54^a^	5.22 ± 0.04	17.84 ± 0.04
	AG/197	134.82 ± 0.36	132.55 ± 0.45	144.81 ± 0.38	24.14 ± 0.22	152.62 ± 1.14^a^	5.23 ± 0.03	17.88 ± 0.03
	GG/79	133.51 ± 0.54^b^	131.13 ± 0.70^b^	143.83 ± 0.54^b^	23.56 ± 0.30^b^	147.42 ± 1.61^b^	5.13 ± 0.04	17.87 ± 0.04
	P value	0.007	0.026	0.024	0.043	0.004	0.147	0.640
a		1.105	1.175	1.000	0.510	3.825	0.045	-0.015
d		0.205	0.245	-0.020	0.070	1.375	0.055	0.025
ɑ		1.125	1.199	0.998	0.517	3.957	0.050	-0.013

Note: Phenotypic values are shown as Means ± SE. Values with the same superscript or no superscript in the same column indicate no significant difference. Values with different superscripts in the same row are significantly different (p < 0.05).

### The additive effects, dominant effects, and substitution effects

The results of the additive effects (a), dominant effects (d), and substitution effects (ɑ) of alleles on phenotypic traits in Dezhou donkeys are shown in Table 3. The additive effects of the A allele on the C allele increased body height, body length, thoracic girth, hide weight, and carcass weight by 1.105 cm, 1.175 cm, 1.000 cm, 0.51 kg, and 3.825 kg, respectively. The dominant effects increased to 0.205 cm, 0.245 cm, 0.070 kg, and 1.375 kg in body height, body length, hide weight, and carcass weight, respectively, but there was a decrease of 0.020 cm in thoracic girth. The substitution effects increased body height, body length, thoracic girth, hide weight, and carcass weight by 1.125 cm, 1.199 cm, 0.998 cm, 0.517 kg, and 3.957 kg, respectively, indicating that the G allele had a negative effect on phenotypic traits in Dezhou donkeys.

## Discussion

With the continuing transfer of traditional farming modes to agricultural mechanization modes, the service function of donkeys is weakening, resulting in the declining economic benefits of donkeys, which has caused the stock of donkeys in China to decrease [27]. Therefore, the conservation and improvement of donkey germplasm resources and their economic efficiency have become the focus of breeding and local breed conservation efforts. Currently, candidate genes are of great importance for improving economically essential traits in Dezhou donkeys and as an effective research tool to study the correlation between genotype and phenotype [12, 13]. Gene mutation sites were discovered to be extremely valuable for developing high-quality breeding donkey resources with a high meat yield [10]. Using high-depth resequencing methods, we screened and identified a SNP associated with body size, hide weight and carcass weight in a population of 393 Dezhou donkeys. This study was the first to investigate the association between genetic diversity of the *NKX1-2* gene and body size traits in Dezhou donkeys.

NKX1-2 is a conserved homeobox gene that encodes a protein containing a homologous structural domain, and the homologous structural domain of the NKX1-2 protein has been well conserved during evolution [28]. NKX1-2 forms and specifies neuronal subpopulations at the early and late stages of vertebrate neural development. Schubert et al. found that the *NKX1-2* gene may be involved in the formation of the posterior neuroectoderm in the embryonic development of mice and is expressed in different regions of the spinal cord and central nervous system later in development, conferring certain specific functions to subsets of neurons [29]. Spann et al. found expression of the *NKX1-2* gene in the spinal cord of the chick embryo and autoregulation in the spinal part of the neural plate [30]. Meanwhile, the *NKX1-2* gene regulates mesoderm formation during zebrafish embryonic development [19]. From the data in Table 3, it can be seen that the mutation at the g.54704925 A > G locus results in GG genotype results for all phenotypes except for the number of thoracic vertebrae, which are lower than those for the AA genotype. Shen et al. used yeast as a model and found that over 3/4 of synonymous mutations have biological significance by affecting the mRNA levels of mutated genes and exerting harmful phenotypic effects on yeast [31]. Therefore, we put forward that the GG genotype is a recessive genotype for all phenotypes except for the number of thoracic vertebrae. This finding has some correlation with previous studies.

Production performance is an essential indicator for breeding, and carcass weight is a significant growth and economic trait. As seen in Table 3, the AA genotype was higher than the GG genotype in all phenotypic data except for the number of thoracolumbar vertebrae, which indicates that the AA genotype is the dominant genotype and can be used to improve growth performance. In this study, it was also found that in both phenotypes, body height and carcass weight, the AA genotype was significantly higher than the GG genotype (P < 0.01), so we can speculate whether the increase in carcass weight was influenced by body height. According to previous studies, the expression of the NKX1-2 gene is regulated by fibroblast growth factor (FGF) signaling factors [32, 33]. The FGF signaling pathway plays an important role in skeletal development, promoting the proliferation and differentiation of osteoblasts, thereby promoting bone growth and repair [34–37]. FGF2 also promotes the proliferation and differentiation of muscle stem cells, thereby promoting muscle growth and repair [38, 39]. In addition, the FGF signaling pathway is also involved in skin development [40]. Therefore, we speculate that the *NKX1-2* gene may be regulated by the FGF signaling pathway in relation to the growth traits of Dezhou donkeys, and the specific mechanism of action still requires further research.

The heterozygosity and effective allele number can objectively reflect the genetic variation of the population, the larger the value, the greater the genetic variation will be. Although the mutation of the codon did not cause the change of amino acid (Gly), it can be seen from Fig. 3 that GGG is a high-frequency codon and GGA is a low-frequency codon. However, if a genotype is too dominant, it may also lead to a decrease in genetic diversity, reducing the adaptability of the entire population and thus affecting the evolution of the entire population. It indicates that the expression of the NKX1-2 gene may be affected by g.54704925 A > G, although further research is still required to determine the precise mechanism. Table 2 shows that the PIC value was 0.373, showing a moderate polymorphic locus (0.25 < PIC < 0.5). It indicates that the population has a high level of polymorphism and that the g.54704925 A > G locus is genetically stable in Dezhou donkeys [41]. The Ho and He values were 0.505 and 0.495, respectively, indicating that the population was rich in genetic diversity [42]. We concentrate on only the correlation between genotype and phenotype, however, the sample size was minimal because our experimental animals came from the same farms with the same husbandry guidelines, management procedures, etc. It resulted in some limitations in our findings and the requirement to increase the study animal sample size.

In general, g.54704925 A > G was significantly correlated with body size traits and carcass weight in Dezhou donkeys and can be used as an assistant marker site for selecting superior individuals in the breeding process, while the *NKX1-2* gene can be used as a candidate gene for breeding meat-type Dezhou donkeys.

## Conclusions

In this study, the *NKX1-2* gene of the Dezhou donkey was examined by high-depth resequencing, and an SNP was found on the exon4. The results showed that the g.54704925 A > G site of the *NKX1-2* gene was significantly associated with body length, thoracic girth, and hide weight (*P* < 0.05), while it was highly significantly associated with body height and carcass weight (*P* < 0.01) in Dezhou donkeys. Therefore, g.54704925 A > G can be used as a marker site for Dezhou donkey selection, and the *NKX1-2* gene is used as a candidate gene for the subsequent selection of meat-type Dezhou donkeys.

### Electronic supplementary material

Below is the link to the electronic supplementary material.

Supplementary Material 1

## Data Availability

The datasets generated during the current study are available in the SRA repository; the persistent web link is https://www.ncbi.nlm.nih.gov/sra/PRJNA950183, and the BioProject accession number is PRJNA950183.

## Declarations

### Manuscript statement

The study was reported in accordance with the ARRIVE guidelines.
